# Delayed Lysis Time at High Multiplicities of Particles in a Chlorovirus-*Chlorella* Interaction

**DOI:** 10.1264/jsme2.ME22068

**Published:** 2022-12-17

**Authors:** Eva J. P. Lievens, Manuela Spagnuolo, Tom Réveillon, Lutz Becks

**Affiliations:** 1 Aquatic Ecology and Evolution Group, Limnological Institute, University of Konstanz, Mainaustraße 252, 78464 Konstanz, Germany

**Keywords:** phenotypic plasticity, phycodnavirus, multiplicity of particles (MOP), multiplicity of infection (MOI), one-step growth curve

## Abstract

When viruses infect microbial cells, their phenotypes depend on the host’s genotype and on the environmental conditions. Here we describe such an effect in laboratory strains of the chlorovirus PBCV-1 and its algal host *Chlorella variabilis*. We studied the growth of six virus isolates, and found that the mean lysis time was 1.34±0.05 times longer at multiplicity of particles (MOP) 10 than at MOP 1. We could not detect any associated changes in burst size. This is a novel plastic trait for chloroviruses, and we hypothesize that it is caused by our specific laboratory algae.

Host-virus interactions play an important role in microbial ecosystems, but their impact is context-dependent (DeLong *et al.*, 2022). For any given virus genotype, traits like lysogeny probability, lysis time, and burst size can vary across host genotypes (Kosznik-Kwaśnicka *et al.*, 2020), abiotic environments (Van Etten *et al.*, 1983; Clasen and Elser, 2007; Cheng *et al.*, 2015; Kosznik-Kwaśnicka *et al.*, 2020), and virus densities (Yoshida *et al.*, 2006; Leggett *et al.*, 2013; Brown and Bidle, 2014; Erez *et al.*, 2017). These plastic responses subsequently affect the virus’ population growth and persistence, as well as its effects on the host population (Choua and Bonachela, 2019).

Recently, we observed a novel plastic phenotype in a well-studied virus-host system. We studied laboratory strains of the chlorovirus PBCV-1, a lytic double-stranded DNA virus, and its host *Chlorella variabilis*, a unicellular freshwater alga. In contrast to previously characterized chlorovirus-host interactions (Van Etten *et al.*, 1983) (Lievens *et al.*, 2022. Life history diversity and signals of trade-offs in a large group of chloroviruses. *bioRxiv*. https://doi.org/10.1101/2022.03.13.484168), we found that the time until lysis consistently depended on the initial virion to host cell ratio, *i.e.* on the multiplicity of particles (MOP). Here we describe these effects and investigate them further.

In all experiments, we used a laboratory clone of *C. variabilis* strain NC64A, hereafter called the ‘lab alga’. This clone was used as the ancestor for the coevolution experiment described in Retel *et al.* (2019, see below), and was kept in batch culture for ≥1 year prior to the present experiments. Algae were cultured at 20°C under constant light and shaking (orbital diameter of 10‍ ‍mm and frequency of 120‍ ‍rpm). We used a modified version of Bold’s Basal Medium (Nichols and Bold, 1965), with ammonium chloride substituted for sodium nitrate as a nitrogen source and double the concentration of trace element solution 4 (first used by Frickel *et al.*, 2016). Algae were in the late exponential phase for all assays (~2×10^6^‍ ‍cells‍ ‍mL^–1^ in our medium). Cultures also contained low levels of bacteria.

We used virus isolates PBCV-1-RK-C6, -D6, -E5, -M2, -N1, and -O2. These isolates were obtained from the coevolution experiment described in Retel *et al.* (2019), in which PBCV-1 and *C. variabilis* NC64A were inoculated into three replicate chemostats (continuous cultures) and tracked for 100 days. In a follow-up experiment, we isolated 85 virus genotypes from different time points of the coevolution experiment, and measured their one-step growth curves at a range of MOPs (assay described below). To our surprise, growth was delayed at higher MOPs for many of the virus isolates, including the ancestral PBCV-1 (unpublished data). Since this had not been reported for other chlorovirus-host interactions (Van Etten *et al.*, 1983) (Lievens *et al.*, 2022. *bioRxiv*. https://doi.org/10.1101/2022.03.13.484168), we selected six isolates with clear delays for further study: PBCV-1-RK-C6, -D6, and -E5 from chemostat replicate I, and -M2, -N1, and -O2 from chemostat replicate III in Retel *et al.* (2019). All virus isolates were in ‘filtrate’ form, *i.e.* a lysed host population filtered through 0.45‍ ‍μm. Filtrates were stored at 4°C in the dark. Their virion concentrations were measured by flow cytometry as described in Lievens *et‍ ‍al.* (2022) (*bioRxiv*. https://doi.org/10.1101/2022.03.13.484168) and published on protocols.io (Lievens, 2022a). Since not all virions measured by flow cytometry are infectious, we describe variations in the filtrate volumes as affecting MOP instead of the multiplicity of infection (MOI).

Experiment 1: To examine the delay in viral growth in more detail, we phenotyped the selected virus isolates following the modified one-step growth (mOSG) assay described in Lievens *et al.* (2022) (*bioRxiv*. https://doi.org/10.1101/2022.03.13.484168) and published on protocols.io (Lievens, 2022b). Briefly, virions and host cells were mixed at an MOP of 0.5, 1, 5, 10, or ≤20, given 15 min to adsorb, and diluted 1:1,000 to synchronize infection. We then tracked the accumulation of progeny virions every 2‍ ‍h for 20 h, with virion concentrations measured by flow cytometry. We also included a parallel 1:10,000 dilution that was sampled after 20 h. Comparing the results of the 1:1,000 and 1:10,000-diluted samples after 20 h allowed us to identify whether secondary infection had occurred after the 1:1,000 dilution (Fig. S1). Time points with suspected secondary infections were removed from the dataset (see below).

The mOSG assay resulted in five growth curves per virus (one per MOP), from which we extracted information as follows. We modeled the virion concentration over time as the sum of unadsorbed virions plus the release of progeny virions:

$$V(t)=\frac{1}{\delta }*\left(\underset{\text{unadsorbed virions}}{\underbrace{A_{a}*M*e^{–k*A_{a}*t_{a}}}}+\underset{\text{release of progeny virions}}{\underbrace{A_{a}*F\left(t;\mu_{l,M},\sigma_{l}^{2},\alpha \right)*b_{M}}}\right)$$

 (Eq. 1),

which is a simplified version of Eq. 1 in Lievens* et al.* (2022) (*bioRxiv*. https://doi.org/10.1101/2022.03.13.484168). *V*(*t*) is the concentration of free virions over time, *δ* is the dilution factor, *A*_a_ is the concentration of algal cells during the adsorption period, and *M* is the MOP. The concentration of unadsorbed virions is calculated from the duration of the adsorption period *t*_a_, the adsorption constant *k*, and the initial virion concentration *A*_a_×*M* (Hyman and Abedon, 2009, Eq. 18.2). The release of progeny virions over time depends on the proportion of lysed cells over time and the burst size per lysed cell. Burst size is expected to vary across cells (Timm and Yin, 2012), but in the absence of single-cell information we could only consider the average burst size *b*. The average burst size was allowed to vary by MOP (*b_M_*) in order to accommodate differences in the proportion of infected cells and any potential effects of delayed lysis on burst size (Goldhill and Turner, 2014). Under the simplification that all host cells produce *b_M_*, the proportion of lysed cells over time could be approximated by the cumulative distribution function *F*(*t*) of a truncated normal distribution with mean lysis time *μ_l_*, standard deviation *σ_l_*, and truncation value *α*. We fit a different *μ_l_* for each MOP (*μ_l,M_*) while keeping *σ_l_* and *α* constant. The truncated normal distribution with constant *σ_l_* was well supported by the data for MOPs 5, 10, and ≤20 (Fig. 1). For MOPs 0.5 and 1, secondary infection obliged us to remove the data from time points 16–20 h; therefore we could not observe the right tails of the lysis time distributions (Fig. 1, right column). The assumption that these tails also followed a truncated normal distribution with the same *σ_l_* was parsimonious, fit the data well, and was supported by comparing our model with models that allowed *σ_l_* to vary with MOP (data not shown). We fit the model to our data using non-linear least squares fitting (function ‘nls’ in base R version 3.6.1, R Core Team, 2014). Function *F*(*t*) was calculated with the EnvStats package (Millard, 2013). Data were *ln*-transformed, outliers were removed before fitting, and parameters were given ample lower and upper bounds. To ensure a robust fit, we ran the model with a range of initial parameters. The model was run separately for each viral strain.

All six virus isolates had a longer lysis time at higher MOPs in experiment 1 (Fig. 2). The mean lysis time *μ_l_* was 1.34±0.05 times longer at MOP 10 than at MOP 1, corresponding to a delay of 2.9±0.4‍ ‍h (mean±SD). There was no consistent increase in mean lysis time from MOP 0.5 to MOP 1. The mean lysis time markedly increased from MOP 1 to MOP 10, and appeared to saturate towards MOP ≤20. The estimates for all parameters are provided in Table S1.

Experiment 2: In experiment 1, we adjusted MOP by varying the volume of filtrate added to the host cells (*e.g.* 1‍ ‍μL of filtrate in 49‍ ‍μL of medium for MOP 0.5; 2‍ ‍μL of filtrate in 48‍ ‍μL of medium for MOP 1). This prompted us to ask whether the longer lysis time at higher MOPs was caused by virions or by another product in the filtrate (*e.g.* signaling molecules, Erez *et al.*, 2017). To test this, we repeated the mOSG assay using two treatments: one treatment was performed as before, while the other used <0.1‍ ‍μm filtrate instead of medium. The <0.1‍ ‍μm filtrate was obtained by filtering the filtrate through 0.1 μm, thereby removing bacteria and chloroviruses (diameter ~0.19‍ ‍μm). In this way, the second treatment maintained a constant volume of elements in the <0.1-μm fraction while varying MOP. We applied these treatments to two representative virus isolates, PBCV-1-RK-D6 and -M2. The mOSG assay was performed as in experiment 1, except that sampling was restricted to 14‍ ‍h. We applied the same ana­lysis to the data, except that the average burst size per host cell (*b*_M_) was given an upper bound of 1.2× the maximum observed burst per host cell. For comparison, in experiment 1 the maximum observed burst per host cell was ≤1.17x higher at time point 20‍ ‍h than at 14‍ ‍h.

Experiment 2 showed that lysis time plasticity was caused by elements in the virion size class (0.1–0.45‍ ‍μm). The mean lysis time *μ_l_* was 1.48±0.04 times longer at MOP 10 than at MOP 1 (mean±SD) regardless of the volume of <0.1-μm filtrate (Fig. 2). In all cases, the model fit at MOP 20 converged on the upper bound for *b* (marked in gray in Fig. 2), suggesting that the average burst size and mean lysis time were overestimated for this MOP. The estimates for all parameters are provided in Table S2.

Experiment 3: Finally, we tested if the change in lysis time was associated with a change in burst size (Goldhill and Turner, 2014). We measured burst size at the single-cell level after exposure to MOP 1 and 10 for virus isolates PBCV-1-RK-D6 and -M2. The experiment was similar to that described by Timm and Yin (2012). Virions and host cells were mixed at an MOP of 0, 1, or 10 under the same conditions as the mOSG assay, given 15 min to adsorb, and diluted to a concentration of 25 cells L^–1^. We then aliquoted 40‍ ‍μL of the diluted solutions into 24 (MOP 0), 368 (MOP 1), and 176 (MOP 10) wells of flat-bottomed tissue culture plates. Following the Poisson distribution, we expected 37% of these wells to contain 1 cell. To identify single-cell wells, plates were centrifuged at 1,258 rcf with slow acceleration for 3‍ ‍min, causing the cells to settle to the bottom, and the bottoms of the wells were imaged using an ImageXpress Micro High Content microscope (Molecular Devices). Wells were imaged based on their autofluorescence in the Cy5 range after 100 ms of light exposure, at 10x magnification. Imaging was completed within 1‍ ‍h of the end of the adsorption period, *i.e.* before the beginning of lysis. Plates were then left under standard culture conditions until 24‍ ‍h after the end of the adsorption period, which provided ample time for lysis (see experiment 1). After 24 h, the plates were transferred to 4°C and stored for 2–3 days. During this time, we used the images to identify wells containing a single cell. Cells were recognized using the MetaXpress software (Molecular Devices), and the software’s identification of single-cell wells was confirmed by a manual inspection of the images. The virion concentration of single-cell wells was measured by flow cytometry (see above).

To analyze the data from experiment 3, we considered the virion concentration in each single-cell well to be drawn from one of three distributions: a truncated lognormal ‘noise’ distribution if the cell was uninfected or the infection was unsuccessful; a lognormal, gamma, Weibull, or truncated normal ‘burst’ distribution (whichever best fit the data) if the infection was successful at MOP 10; and the same ‘burst’ distribution with different parameters if the infection was successful at MOP 1:

$$V_{0}\;\sim \;\text{TruncatedLognormal}(\mu_{n},\sigma_{n}^{2},0.1)$$

 (Eq. 2.1)

$$V_{1}\;\sim \;\left(1–p_{b,1}\right)\times \text{TruncatedLognormal}(\mu_{n},{\sigma_{n}}^{2},0.1)$$

$$+p_{b,1}\times \text{Lognormal}(\mu_{b,1},{\sigma_{b,1}}^{2})$$

 (Eq. 2.2)

$$V_{10}\;\sim \;\left(1–p_{b,10}\right)\times \text{TruncatedLognormal}(\mu_{n},{\sigma_{n}}^{2},0.1)$$

$$+p_{b,10}\times \text{Lognormal}(\mu_{b,10},{\sigma_{b,10}}^{2})$$

 (Eq. 2.3)

(for the case of lognormal burst distributions). *V* represents the virion concentration in single-cell wells, *p* is the probability of successful infection, *μ* and *σ* represent the mean and standard deviation of the noise (subscript *n*) and burst distributions (subscript *b*), respectively, and 0.1 is the truncation value. Subscripts 0, 1, and 10 identify the MOP treatments. We fit these mixture distributions to the data using maximum likelihood optimization (likelihoods for the truncated distributions calculated with the EnvStats package, Millard, 2013; optimization function ‘optim’ in base R version 3.6.1, R Core Team, 2014). The effect of MOP on burst size was then tested by comparing the full model (*μ_b_*_,1_≠*μ_b_*_,10_ and *σ_b_*_,1_≠*σ_b_*_,10_) to a reduced model (*μ_b_*_,1_≠*μ_b_*_,10_ and *σ_b_*_,1_=*σ_b_*_,10_) and null model (*μ_b_*_,1_=*μ_b_*_,10_ and *σ_b_*_,1_=*σ_b_*_,10_) using likelihood ratio tests. This procedure was performed separately for D6 and M2. Data were transformed by the addition of 0.1 to allow lognormal fitting of the noise. All models were run with a range of initial parameters, and the parameters were given ample lower and upper bounds.

There was no detectable effect of MOP on burst size in experiment 3. Both virus isolates produced highly variable burst distributions (Fig. 3). For PBCV-1-RK-D6, there were 12, 113, and 41 confirmed single-cell wells at MOPs 0, 1, and 10, respectively. The burst distributions were given a truncated normal shape, but there were not enough successfully infected cells at MOP 1 to robustly fit the full and reduced models. Thus we can only report the null model, which was an acceptable match for the data. The null model predicted a median noise measurement of 0.2 and a median burst measurement of 8.2, which corresponded to a median burst size of 1,604 virions per successful infection (Fig. 3). The full parameter estimates were: *p*_1_=0.12, *p*_10_=0.72, *μ_n_*=–‍70.1, *σ_n_*=9.6, *μ_b_*_,1_=*μ_b_*_,10_=7.9, and *σ_b_*_,1_=*σ_b_*_,10_=5.0. For PBCV-1-RK-M2, there were 9, 110, and 54 confirmed single-cell wells at MOPs 0, 1, and 10, respectively. The burst distributions were given a lognormal shape. There was no significant effect of MOP on the standard deviation of the burst distribution (full vs. reduced model, χ^2^[1]=0.26, *P*=0.61), nor on its mean (reduced vs. null model, χ^2^[1]=0.27, *P*=0.60). The null model predicted a median noise measurement of 0.2 and median burst measurement of 8.9, which corresponded to a median burst size of 1,740 virions per successful infection (Fig. 3). The full parameter estimates were: *p*_1_=0.20, *p*_10_=0.81, *μ_n_*=–1.9, *σ_n_*=1.5, *μ_b_*_,1_=*μ_b_*_,10_=2.2, and *σ_b_*_,1_=*σ_b_*_,10_=0.6.

Overall, we have demonstrated that MOP has a plastic effect on lysis timing in these chlorovirus-host combinations (Fig. 2). More specifically, lysis time increased when there was a higher concentration of filtrate elements in the size class 0.1–0.45‍ ‍μm (Fig. 2). This size class includes both virions and bacteria, but two findings refute bacteria as causative elements. First, the bacterial concentrations in the filtrates differed by an order of magnitude in experiments 1 and 2 (measured by flow cytometry, Table S3), yet the MOP effects were similar. Second, bacteria were present in cases where we did not observe plastic lysis times (Fig. S2A and Table S3). Therefore, we conclude that lysis time plasticity is related to changes in the surrounding virion concentration.

Lysis time plasticity is a virocell trait that can be influenced by the environment, viral genetics, host genetics, and their interactions (DeLong *et al.*, 2022). Based on a comparison with the findings reported by Lievens *et al.* (2022) (*bioRxiv*. https://doi.org/10.1101/2022.03.13.484168), we hypothesize that plasticity is a consequence of host genetics in this case. Lievens *et al.* did not observe plasticity in PBCV-1 infecting *C. variabilis* NC64A. The experimental conditions were nearly identical in the two studies, arguing against an environmental difference. It is also unlikely that the viral genetics were substantially different: Lievens *et al.*’s PBCV-1 was separated by ~5 passages from Retel *et al.*’s ancestral PBCV-1, which also displayed plasticity when tested in our lab alga (Fig. S2B). In contrast, Lievens *et al.* used a host clone from the University of Nebraska-Lincoln collection (UNL), whereas the lab alga had been cultured under our lab conditions for more than one year. This may have led to genetic differences between the clones. We thus hypothesize that lysis time plasticity is due to an evolved change in the lab algal clone. This interpretation is supported by experiments with different Retel *et al.* virus isolates, in which plasticity did not appear in the UNL alga (Fig. S2). It is important to note that our lab alga was cultured without viruses, so lysis time plasticity would not have evolved as an adaptation to viral presence.

Interestingly, we could not detect any effect of delayed lysis on burst size. Our direct experimental results (Fig. 3) were supported by an ana­lysis of the fitted parameters from experiment 1: we compared the fitted *b_M_*~MOP with the expected *b_M_*~MOP given a constant burst size, and found an excellent match between the two (Fig. S3). This contradicts the common expectation that delayed lysis provides more time for progeny virus production, resulting in a higher burst size (Goldhill and Turner, 2014). Since burst size is typically measured in infectious virions (*e.g.* plaque-forming units) instead of virions, this discrepancy could be resolved if early-released virions are less infectious. Understanding the cellular mechanisms underlying lysis time plasticity may also provide insights into the contradiction. The contrasting expectation that high MOPs lead to declines in burst size was also unsupported (Van Etten *et al.*, 1983; Brown and Bidle, 2014).

Chlorovirus-host interactions are known to depend on various aspects of the abiotic environment and host state (Van Etten *et al.*, 1983; Clasen and Elser, 2007; Cheng *et al.*, 2015; Horas *et al.*, 2018), but we believe this is the first study to show that their lysis time can respond to viral density. In contrast to e.g. bacteriophage systems, in which plasticity in lysis time or temperateness is an adaptive virus trait (Leggett *et al.*, 2013; Goldhill and Turner, 2014), plastically delayed lysis appears to be an incidental trait of our lab algal clone. Nevertheless, the laboratory emergence of this phenotype suggests that it can also occur in natural *C. variabilis*, where it would have the effect of slowing chlorovirus epidemics. Future studies on this trait should confirm that lysis time plasticity is host-associated, investigate the underlying mechanism, and explore the potential consequences for chlorovirus ecology.

## Citation

Lievens, E. J.. P.., Spagnuolo, M., Réveillon, T., and Becks, L. (2022) Delayed Lysis Time at High Multiplicities of Particles in a Chlorovirus-*Chlorella* Interaction. *Microbes Environ ***37**: ME22068.

https://doi.org/10.1264/jsme2.ME22068

## Supplementary Material

Supplementary Material

## Figures and Tables

**Fig. 1.**
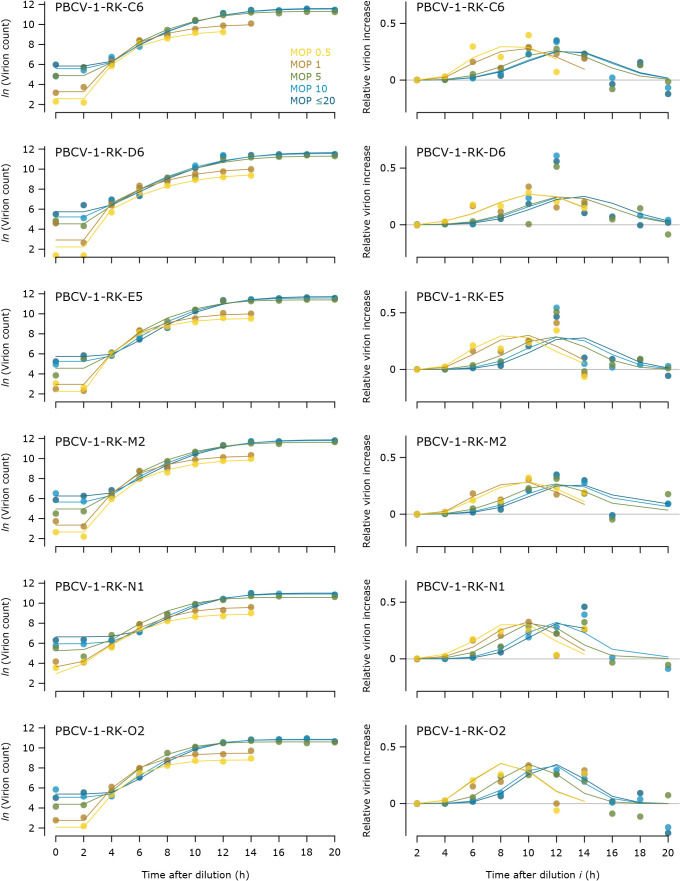
Model fitting for experiment 1. Points represent modified one-step growth (mOSG) data after outliers and time points with suspected secondary infection were removed (see the main text). Lines represent model predictions. Left column: The accumulation of virions over time for each multiplicity of particles (MOP). Right column: The accumulation of virions represented as a relative increase, which is calculated for each MOP as (increase in the virion count from time point *i*-1 to *i*)÷(maximum virion count). Note that this calculation increases noise in the observed data because it compounds the measurement error at time points *i*-1 and *i*.

**Fig. 2.**
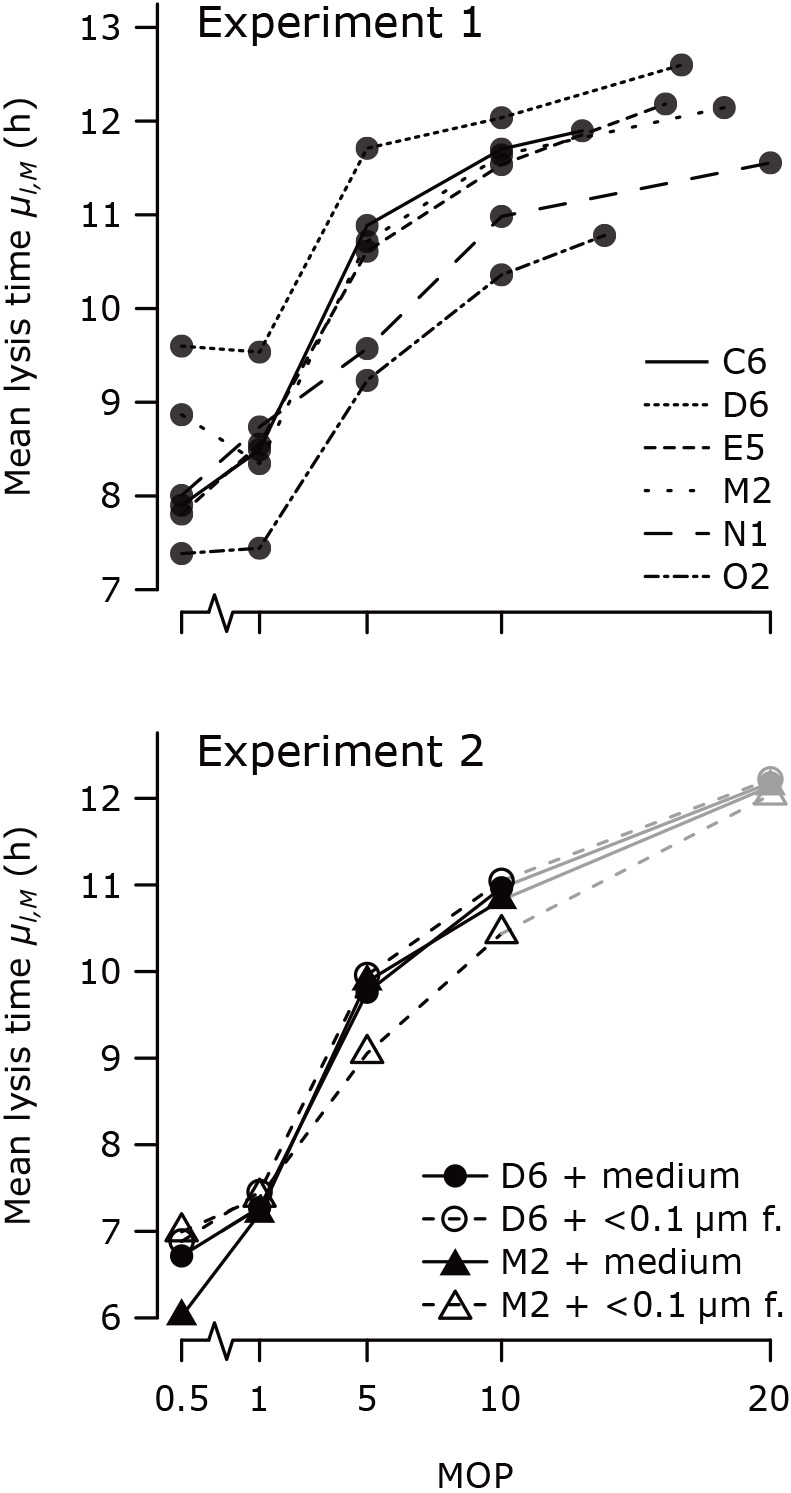
Mean lysis times increased with multiplicity of particles (MOP) in experiments 1 and 2. Each point represents a fitted mean lysis time *μ_l,M_*; lysis times for the same virus isolate or virus isolate + treatment are connected by a line. Points and line segments colored in gray indicate a poor fit for *μ_l,M_* (see the main text). Virus isolates are abbreviated to their last two characters (*e.g.* ‘C6’ is PBCV-1-RK-C6); ‘<0.1‍ ‍μm f.’: <0.1‍ ‍μm filtrate.

**Fig. 3.**
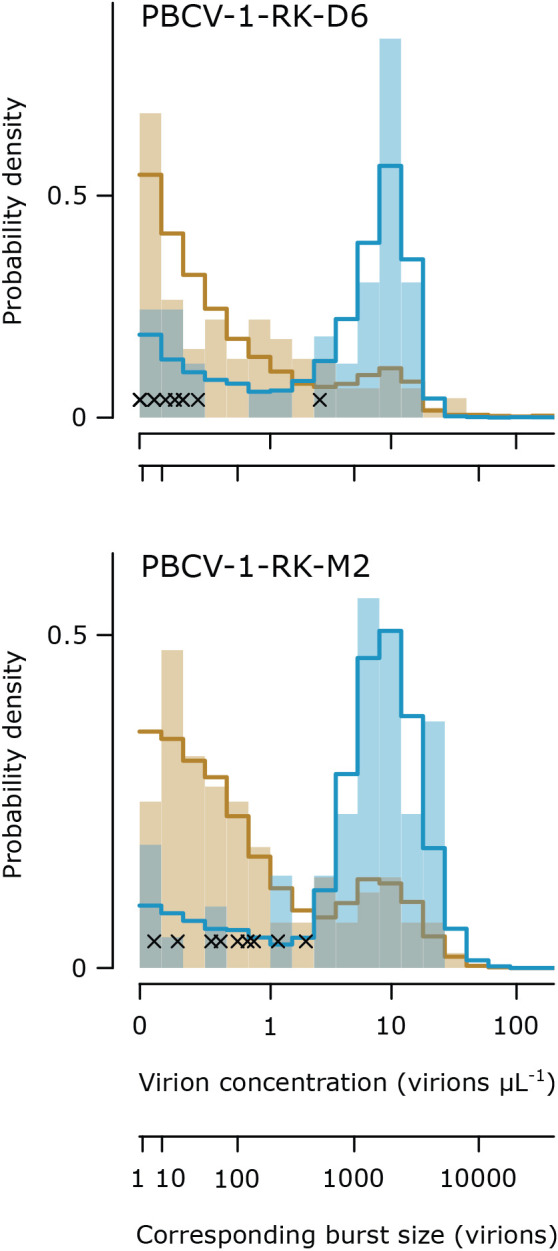
Multiplicity of particles (MOP) did not detectably affect burst size in experiment 3. Histograms show virion concentrations measured in single-cell wells after exposure to MOP 1 (orange) and MOP 10 (blue); crosses mark the virion concentrations in single-cell wells after exposure to MOP 0. Lines show model predictions for the null models, based on 10,000 simulations per MOP. The predicted distributions are dominated by noise (values between 0 and ~1 virion μL^–1^) at MOP 1 and by bursts (values above ~2 virions μL^–1^) at MOP 10. The two x-axes are on the *ln* scale, and indicate the virion concentration as measured by flow cytometry (data +0.1-transformed) and corre­sponding burst sizes per single cell (virion concentrations ×5-fold dilution for flow cytometry ×40‍ ‍μL volume).
