# Supplementary material for: Delayed Lysis Time at High Multiplicities of Particles in a Chlorovirus-Chlorella Interaction
Source: Microbes Environ. 2022 Dec 17;37(5):ME22068. doi: 10.1264/jsme2.ME22068 (PMC9763037; doi:10.1264/jsme2.ME22068)
Supplement: Supplementary file 1 — Supplementary Material [file 37_22068_s1.pdf]

Supplementary Material

Table S1. Parameter estimates for experiment 1.

virus isolate	MOPs tested	k (×10^{-9})	$\mu_{l,M}$ (h)	σ_l (h)	α (h)	b_M (virions)
PBCV-1-RK-C6	0.5, 1, 5, 10, 13.0	6.2	7.9, 8.5, 10.9, 11.7, 11.9	3.0	3.4	111, 204, 748, 950, 1007
PBCV-1-RK-D6	0.5, 1, 5, 10, 16.7	7.8	9.6, 9.5, 11.7, 12.0, 12.6	3.1	2.0	123, 220, 751, 971, 1068
PBCV-1-RK-E5	0.5, 1, 5, 10, 16.1	7.7	7.8, 8.6, 10.6, 11.5, 12.2	2.7	3.4	127, 206, 819, 994, 1123
PBCV-1-RK-M2	0.5, 1, 5, 10, 18.3	5.9	8.9, 8.3, 10.7, 11.6, 12.1	2.9	3.4	182, 262, 1016, 1227, 1305
PBCV-1-RK-N1	0.5, 1, 5, 10, 20	4.6	8.0, 8.7, 9.6, 11.0, 11.6	2.4	0.7	66, 134, 361, 505, 552
PBCV-1-RK-O2	0.5, 1, 5, 10, 13.8	8.5	7.4, 7.4, 9.2, 10.4, 10.8	2.2	3.4	61, 120, 370, 455, 474

Table S2. Parameter estimates for experiment 2.

virus isolate	treatment	MOPs tested	k (×10^{-9})	$\mu_{l,M}$ (h)	σ_l (h)	α (h)	b_M (virions)
PBCV-1-RK-D6	+ medium	0.5, 1, 5, 10, 20	5.3	6.7, 7.3, 9.8, 11.0, 12.2	3.1	3.1	110, 235, 890, 1190, 1404
PBCV-1-RK-D6	+ <0.1 μm filtrate	0.5, 1, 5, 10, 20	4.5	6.9, 7.5, 10.0, 11.0, 12.2	3.3	3.2	140, 245, 913, 1396, 1667
PBCV-1-RK-M2	+ medium	0.5, 1, 5, 10, 20	3.3	6.0, 7.2, 9.9, 10.8, 12.1	3.7	3.3	112, 204, 879, 1324, 1658
PBCV-1-RK-M2	+ <0.1 μm filtrate	0.5, 1, 5, 10, 20	6.5	7.0, 7.4, 9.1, 10.4, 12.0	3.3	3.0	113, 244, 854, 1376, 1727

Table S3. Bacterial concentrations in the filtrates, as measured by flow cytometry. Experiments 4-8 correspond to the unpublished data represented in Fig. S2.

experiment	virus isolate	bacterial concentration ($\times 10^6$ cells mL^{-1})	plastic lysis time
1	PBCV-1-RK-C6	4.5	yes
1	PBCV-1-RK-D6	2.9	yes
1	PBCV-1-RK-E5	6.3	yes
1	PBCV-1-RK-M2	3.8	yes
1	PBCV-1-RK-N1	3.4	yes
1	PBCV-1-RK-O2	2.6	yes
2	PBCV-1-RK-D6	22.6	yes
2	PBCV-1-RK-M2	16.8	yes
4 (Fig. S2A)	PBCV-1-RK-A1	1.6	no
4 (Fig. S2A)	PBCV-1-RK-F1	1.7	no
5 (Fig. S2A)	PBCV-1-RK-A1	1.9	no
6 (Fig. S2A)	PBCV-1-RK-A1	0.7	no
6 (Fig. S2A)	PBCV-1-RK-F1	1.0	no
6 (Fig. S2A)	PBCV-1-RK-H4	1.4	no
7 (Fig. S2B)	PBCV-1-RK-A1	2.9	yes
7 (Fig. S2B)	PBCV-1-RK-F1	7.5	no
7 (Fig. S2B)	PBCV-1-RK-H4	3.0	yes
8 (Fig. S2B)	PBCV-1-RK-A1	unknown	yes
8 (Fig. S2B)	PBCV-1-RK-F1	unknown	yes
8 (Fig. S2B)	PBCV-1-RK-H4	unknown	yes

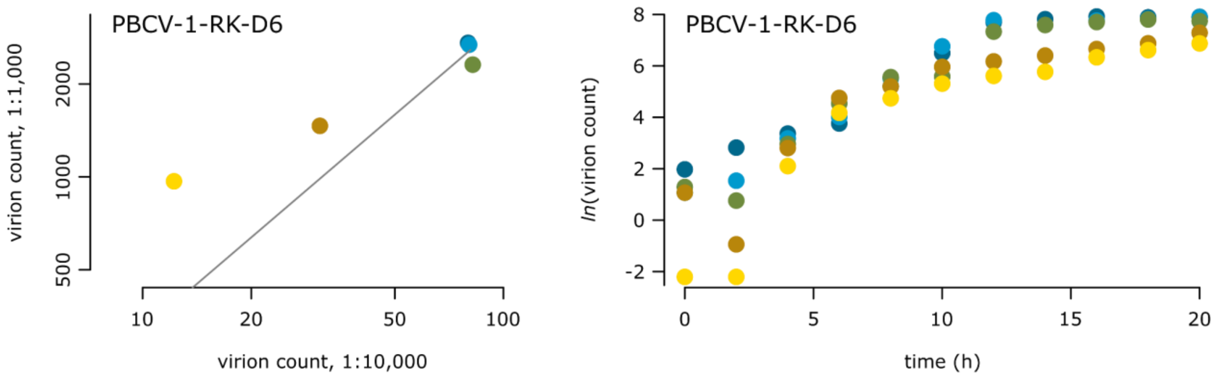

Figure S1. Identification of secondary infection in experiment 1.

Example for virus isolate PBCV-1-RK-D6. Points represent the modified one-step growth (mOSG) data after outliers were removed, colored by MOP (yellow: MOP 0.5, orange: 1, dark green: 5, blue: 10, indigo: 20). **Left:** Comparison of the virion concentrations at 20 h for the 1:1,000- and 1:10,000-diluted samples; axes on the *ln* scale. Secondary infection occurs when the progeny viruses from the ‘primary’ infections (the synchronized infections that started during the adsorption period) adsorb to and infect new host cells. It is very unlikely to occur after dilution by 1:10,000, because adsorption is disproportionately reduced (Hyman and Abedon 2009, Eq. 18.2). Secondary infection is also unlikely at MOPs ≥10, because there are few new host cells available. In the absence of secondary infection, therefore, the virion concentrations in the 1:1,000-diluted samples should be 10 times higher than those in the 1:10,000-diluted samples, plus a potential offset for environmental differences. That baseline expectation is represented by the gray line with slope 10 and intercept determined by the concentrations for MOPs ≥10 (takes any environmental differences into account). If secondary infection did occur, the virion concentrations in the 1:1,000-diluted samples skew upwards at low MOPs. In this example, secondary infection occurred at MOPs 0.5 and 1. **Right:** The secondary infection was also visible in the accumulation of virions over time for each multiplicity of particles (MOP): at time points ≥16 h, there is a clear secondary increase for MOPs 0.5 and 1. These points were removed for the analysis of experiment 1 and in Fig. 1.

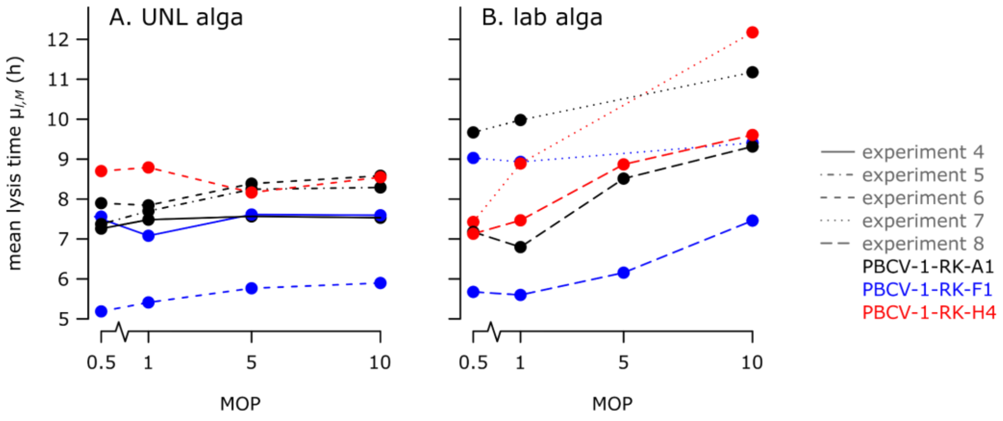

Figure S2. Mean lysis time in the UNL (A) and lab (B) algae.

This graph presents the model fits of various modified one-step growth (mOSG) assays performed on virus isolates PBCV-1-RK-A1, -F1, and -H4. Virus isolate PBCV-1-RK-A1 is the PBCV-1 ancestor used by Retel, Kowallik et al. (2019). The mOSG assays were run as part of different experiments (unpublished data, here called experiments 4 to 8), and are therefore not directly comparable. Each point represents a fitted mean lysis time $\mu_{l,M}$; lysis times for the same virus isolate in the same experiment are connected by a line. Colors identify virus isolates; line types identify experiments.

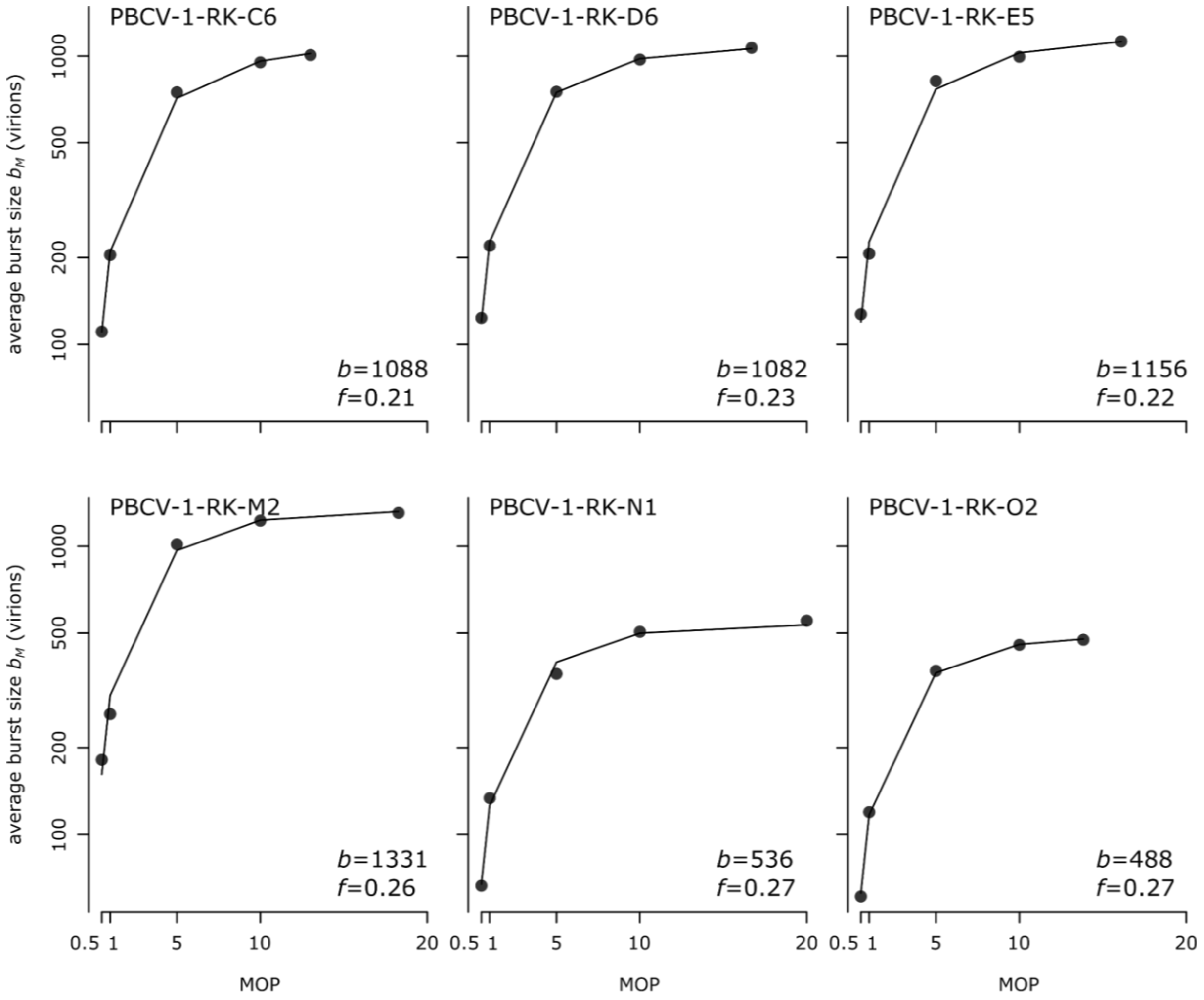

Figure S3. Analysis of MOP effects on burst size, based on experiment 1.

Points represent the fitted average burst sizes b_M from experiment 1 (y-axis on the *ln* scale). Lines represent the fit of a 'constant burst size' model to these points. This model is assumes that burst size does not depend on MOP, and is derived as follows: As MOP increases, the proportion of infected algal cells approaches 1, following a Poisson probability function whose rate depends on the number of virions added (i.e. the MOP) and the fraction of those virions that were able to adsorb to and infect a cell (fraction *f*, which we assume is independent of MOP). If each infected algal cell produces the same burst size, the average burst size per host cell should follow $b_M(M) = (1 - e^{-f*M}) * b$ (the constant burst size model), where *b* is the burst size per infected cell *b*, and *M* is the MOP. We fit this model to the *ln*-transformed b_M data for each virus isolate using nonlinear least squares fitting (function 'nls' in base R version 3.6.1, R Core Team 2014). The fitted parameters for *f* and *b* are shown in the plots.
